# Lower-Limb Amputees and Family Caregivers: Challenges, Needs, and Strategies for Empowerment—A Qualitative Study

**DOI:** 10.3390/nursrep15050166

**Published:** 2025-05-12

**Authors:** Diana Rodrigues, Luís Carvalho, Cristina Pinto

**Affiliations:** 1 RISE-Health, Faculty of Medicine, University of Porto, Alameda Prof. Hernâni Monteiro, 4200-319 Porto, Portugal; luiscarvalho@esenf.pt (L.C.); cmpinto@esenf.pt (C.P.); 2 ICBAS—Abel Salazar Institute of Biomedical Sciences, University of Porto, 4050-313 Porto, Portugal; 3 Nursing School of Porto, 4200-072 Porto, Portugal

**Keywords:** amputees, disabled person, empowerment, family caregiver, hospital to home transition, lower limb, self-care

## Abstract

**Background/Objectives:** Lower-limb amputation profoundly affects individuals and their family caregivers, particularly during home transition after hospital discharge. Understanding the needs, challenges, and emotions during this period is essential for designing effective family centered empowerment interventions. This study aimed to explore the lived experiences of amputees and their caregivers, identify their needs and challenges, and identify strategies to foster empowerment, resilience, and adaptation after amputation. **Methods**: This qualitative, descriptive-exploratory study involved semi-structured interviews with 37 dyads, each comprising an amputee who has undergone major dysvascular lower-limb amputation and their primary caregiver, who provided home care. The participants attended follow-up consultations post-amputation. Data were collected over a 13-month period and analyzed using qualitative content analysis based on Bardin’s methodology, with support from ATLAS.ti 23.3.4 software for coding and data organization. **Results**: Four categories emerged: (i) difficulties faced, including loss of autonomy, mobility challenges, architectural barriers, and emotional strain; (ii) home discharge, emphasizing functional training for amputees and caregivers and the need for community support; (iii) impact of amputation, highlighting acceptance difficulties, psychological distress, social isolation, and lifestyle changes; and (iv) empowerment strategies, focusing on psychological support, skills training, assistive devices, and coordinated care. Tailored interventions such as peer support, home adaptations, and multidisciplinary care are essential for resilience, independence, and improved quality of life. **Conclusions**: Family centered empowerment strategies are vital for improving the outcomes of amputees and caregivers. Interventions that prioritize caregiver education, psychological support, and enhanced accessibility promote resilience, autonomy, and quality of life. These findings highlight the need for integrated hospital-to-community programs.

## 1. Introduction

Limb loss, particularly lower-limb amputation, is a life-altering event that significantly impacts individuals’ physical, psychological, and social well-being, particularly during their transition home after hospital discharge. Dysvascular lower-limb amputations, primarily caused by complications of peripheral arterial disease and diabetes mellitus, remain a significant health concern despite advancements in treatment [1]. The most common cause of lower-limb amputation is dysvascular amputation caused by peripheral arterial disease or diabetes [1]. Peripheral arterial disease (PAD) is a major cause of non-traumatic lower-limb amputations in people with diabetes [2]. The presence of diabetes mellitus significantly increases the risk of developing PAD, accelerates its progression, and exacerbates its severity, thereby increasing the likelihood of limb amputation [3]. Dysvascular lower-limb amputees often experience high multimorbidity, with diabetes, hypertension, phantom limb pain, and musculoskeletal pain being common health conditions [4]. Dysvascular lower-limb amputations significantly affect mobility, social engagement, psychological health, and the overall quality of life. Complications from diabetes, such as these amputations, are a major and increasing contributor to global disability [4,5,6].

Lower-limb amputation (LLA) has a profound impact on patients’ quality of life, affecting the physical, mental, and social dimensions [7]. In addition to physical adaptations, patients have a lower quality of life, body image, and self-esteem and face emotional challenges and concerns about lifestyle changes [7,8]. The transition from inpatient rehabilitation to home can be challenging, highlighting the importance of coping strategies and social support in managing everyday tasks [9].

LLA has a significant impact on all aspects of quality of life (QOL). Age plays a crucial role, and QOL declines as age increases. Amputees using prosthetic devices tend to experience better QOL. Additionally, many amputees have reported experiencing residual limb pain and phantom limb pain [10]. After amputation, the majority of lower-limb amputees experience severe mobility impairments and become financially dependent. The effects of LLA go beyond the individual, impacting families who frequently struggle with shifts in the traditional gendered roles of primary earners [11].

Amputees and their family caregivers face numerous challenges such as rehabilitation, prosthetic adaptation, and mobility limitations. Lower-limb amputees and their caregivers face limited financial and psychosocial support and have to deal with infrastructural barriers to accessing and using prostheses [12]. Major lower-limb amputees face a range of physical, mental, practical, and financial difficulties, all of which require support and understanding to help them adapt to their new reality [7]. Caring for someone who has undergone amputation causes psychological, financial, and physical stress to families. The caregiver burden tends to increase significantly with the severity of the amputation, with more significant amputations creating a heavier strain than minor ones [13].

Implementing family-centered empowerment programs can significantly improve outcomes for both patients and their caregivers. Family-centered interventions can significantly enhance outcomes for family caregivers by reducing burden, improving quality of life, and alleviating stress and depression. Moreover, the family-centered empowerment models have been shown to significantly improve the quality of life of adults with chronic diseases [14,15]. A family-centered empowerment intervention as a FOCUS program increased self-efficacy and improved quality of life for both patients and their caregivers [16].

The transition home following a lower-limb amputation represents a critical period of adjustment that affects not only lower-limb amputees but also their family caregivers. This dyadic experience involves complex physical, emotional, and social transformations as both individuals navigate new roles, responsibilities, and challenges in daily life. Recognizing the interconnected nature of their experiences is essential to fully understand the scope of needs, obstacles, and coping strategies that arise during this phase. Therefore, this study aimed to achieve the following:

✓ To explore the lived experiences of lower-limb amputees and family caregivers during the transition home after hospital discharge.
✓ To identify the needs, difficulties, and challenges faced by lower-limb amputees and family caregivers.
✓ To uncover strategies that promote empowerment, resilience, and adaptation to post-amputation life.

## 2. Materials and Methods

This was a descriptive qualitative study conducted in a vascular surgery unit in northern Portugal. Throughout the research, we adhered to rigorous criteria and followed the Consolidated Criteria for Reporting Qualitative Studies (COREQ) to ensure accurate qualitative reporting.

A single researcher with a nursing background and training in qualitative methods conducted and audio-recorded all interviews. Methodological rigor was ensured by following the Consolidated Criteria for Reporting Qualitative Research (COREQ). Credibility was supported through accurate transcription and prolonged engagement with participants; transferability through detailed contextual and participant descriptions; dependability through regular team discussions on methodological decisions; and confirmability via the use of a reflective journal and team debriefings. These strategies enhanced transparency, reduced bias, and ensured that findings reflected participants’ perspectives.

### 2.1. Participants and Sample

Participants were recruited from a follow-up hemodynamic consultation for vascular diseases at a Vascular Surgery Unit in a hospital in Northern Portugal. The study included dyads consisting of individuals with major dysvascular lower-limb amputation and their primary family caregivers. These individuals were approached during routine consultations, where they were informed about the study’s purpose and invited to participate. The recruitment was carried out using convenience sampling, as participants were selected based on their availability and willingness to engage in the study. All participants were informed of the voluntary nature of their involvement, and written consent was obtained prior to their inclusion. The researchers did not have prior relationships with the participants before the study began.

The study population included individuals from various socio-economic backgrounds, with both amputees and their family caregivers sharing the experiences of navigating the challenges associated with post-amputation care.

The inclusion criteria for amputees were as follows.

(1) Aged 18 years or older;
(2) Having undergone a major lower-limb amputation due to vascular causes;
(3) Living at home;
(4) Receiving assistance with activities of daily living (ADLs) from a family caregiver;
(5) Possessing adequate cognitive and comprehension abilities;
(6) Being at least six months post-amputation.

The inclusion criteria for family caregivers were as follows.

(1) Aged 18 years or older;
(2) Providing care at home for a dysvascular major lower-limb amputee;
(3) Identified as the primary caregiver, responsible for the amputee’s care since hospital discharge;
(4) Assisting lower-limb amputees with activities of daily living.

The exclusion criteria included:

(1) Lower-limb amputees who were independent in all activities of daily living (ADLs);
(2) Lower-limb amputees residing in nursing homes or other institutional settings;
(3) Family caregivers who were not the primary caregivers.

Sampling was conducted over a 13-month period until data saturation was achieved. During the recruitment process, five individuals with lower-limb amputations and five family caregivers declined participation. Additionally, seven potential participants did not meet the inclusion criteria, and eight scheduled follow-up consultations were missed without rescheduling. Given the dyadic nature of the study, the absence or refusal of either member (amputee or caregiver) led to the exclusion of the entire dyad. These instances were accounted for during recruitment, and sampling continued until data saturation was reached.

The sample size was determined based on the principle of data saturation, which occurs when additional interviews no longer yield new information or insights about the phenomenon under study, resulting in redundancy. As Bardin [17] emphasized, researchers must remain vigilant during categorization and analysis to identify when no new elements emerge. In this study, saturation was confirmed after 37 interviews with lower-limb amputees and 37 interviews with their primary family caregivers, ensuring a comprehensive understanding of the participants’ experiences.

### 2.2. Data Collection

Two instruments were used for the data collection: a sociodemographic questionnaire and semi-structured interviews. The sociodemographic questionnaire, tailored for each participant group, gathered information on sex, age, educational level, and employment status at the time of surgery (for amputees). Additional questions included the amputation level (for amputees), employment status at the time of the interview (for family caregivers), and the caregiver’s relationship with the amputee.

The sociodemographic questionnaire was developed by the research team specifically for the objectives of the study. Although it was not pilot tested, the questionnaire was meticulously designed based on a thorough review of the existing literature and expert input to ensure its relevance and clarity. The research team carefully considered the content and structure to ensure that it was suitable for capturing the necessary information and addressing the study’s aims.

Semi-structured interviews were conducted using a guiding framework developed by the research team, based on existing literature on post-amputation recovery and family caregiving. The framework was composed of open-ended questions designed to explore the perceived needs, emotional responses, challenges, lived experiences, and empowerment strategies of lower-limb amputees and their family caregivers following hospital discharge and their transition back home. This approach ensured consistency across interviews while allowing participants the freedom to express their thoughts and experiences in depth.

The lead researcher scheduled home visits to conduct individual interviews with both lower-limb amputees and family caregivers. After informed consent was obtained, the interviews were digitally recorded and transcribed. Each session lasted for approximately 20–25 min per participant. Data collection continued until saturation was reached. The semi-structured interviews were conducted by a single interviewer with a nursing background. The interviewer had experience working with individuals with lower-limb amputations and their caregivers, which was particularly relevant for ensuring comfort and eliciting rich, meaningful responses during the interviews.

All interviews were audio-recorded using a digital voice recorder to ensure accurate capture of participants’ responses. The recordings were transcribed verbatim into text files using Microsoft Word, and the transcriptions were subsequently reviewed and cross-checked by the lead researcher to ensure fidelity to the original recordings. Data collection continued until data saturation was reached—that is, when no new themes, categories, or insights were emerging from subsequent interviews.

### 2.3. Data Analysis and Treatment

In our study, we employed an emergent coding approach, allowing themes to naturally arise from the participants’ narratives. This inductive method enabled us to capture the authentic experiences of lower-limb amputees and their family caregivers, ensuring that the analysis remained grounded in their perspectives. Qualitative content analysis was conducted using Bardin’s methodology [17] and ATLAS.ti 23.3.4 software (ATLAS.ti Scientific Software Development GmbH, Berlin, Germany). Participants’ anonymity was ensured through a coding system consisting of one or two letters followed by a number. The thematic-categorical approach comprises three main phases.

In the pre-analysis phase, researchers conducted a floating reading of the transcripts to gain general understanding and identify preliminary categories, laying the foundation for subsequent analysis.

Exploring the material involved organizing the data in ATLAS.ti, where researchers created codes that represented units of meaning and grouped them into thematic categories. The software facilitated a clear visualization of the relationships between the codes and provided insights into the frequency of themes, thus enhancing the understanding of the content.

In the processing and interpretation phase, the researchers triangulated the data between the two groups of participants, making it possible to identify convergence and divergence. The inferences were generated based on a theoretical framework. Each phase of the analysis was conducted independently by two researchers, with a third researcher being consulted in the event of discrepancies. Finally, through analysis and discussion, researchers reached a consensus on the results. The integration of Bardin’s methodology with ATLAS.ti enables structured and in-depth analysis, leading to significant and well-founded findings.

Researcher bias was considered, as personal perceptions could potentially influence the interpretation of the data. To minimize this effect, qualitative content analysis was conducted collaboratively by multiple researchers, who jointly developed and refined the coding and categorization. This approach ensured triangulation and facilitated collective discussion of various interpretations, ultimately strengthening the validity of the findings. Furthermore, the research team acknowledged their own positionalities, including professional backgrounds and experiences, and reflected on how these factors might influence interactions with participants and data interpretation. Regular team discussions were held to critically examine these influences, enhancing the transparency and trustworthiness of the study.

### 2.4. Ethical Considerations

This study adhered to the ethical principles for research involving human subjects in accordance with the Declaration of Helsinki. Approval was obtained from the hospital’s ethics committee where data collection took place. Each form was coded to ensure confidentiality, and participants provided informed consent. Anonymity was maintained by assigning identifiers: (FC = Family Caregiver) and (A = Amputee), followed by a sequential interview number.

## 3. Results and Discussion

### 3.1. Characterization of Amputees

The study included 37 participants with dysvascular major lower-limb amputation, comprising 81% men and 19% women, each paired with a corresponding family caregiver, forming 37 dyads. The majority were over 65 years old (75.7%), with the most represented age group being 75–80 years, accounting for 27% of the sample. Most amputees were male, as evidenced by a study in which 80.5% of the participants were men. Age also plays a significant role, with incidence rates rising sharply among older individuals [4,18]. A significant portion of the participants had a low level of education, with 70% having completed only four years of basic schooling. At the time of surgery, 54% were retired, and only 5% were employed. Studies suggest that these patients have lower education levels than the general population [19]. In addition, the majority were retired or unemployed at the time of amputation, with only a small proportion actively employed [20]. Regarding the type of amputation, 75.7% of the patients underwent transfemoral (above-the-knee) amputation. Above-knee amputations are associated with a higher degree of disability and morbidity than below-knee amputations [21]. A detailed characterization of the amputee participants, including sociodemographic and clinical data, is provided in Table S1.

### 3.2. Characterization of Family Caregivers

The study included 37 dyads, with family caregivers serving as the primary caregivers for individuals with dysvascular major lower-limb amputations, providing assistance with daily living activities at home. A total of 37 family caregivers were involved, with women comprising 81% of the caregivers. The majority (65%) were over 60 years old, with the largest age group being 66–70 years, representing 29.7% of participants. In terms of education, 46% had completed four years of basic education. At the time of the study, 40.5% of participants were employed. Family caregivers of older adults experience considerable challenges, with women making up the majority of caregivers according to multiple studies [22,23]. Many of these caregivers are also older, with a significant proportion being over the age of 60 [23]. Although education levels differ, lower educational attainment is linked to higher caregiver burden [23]. Regarding their relationships with amputees, 65% were spouses or partners. A detailed characterization of the family caregivers, including sociodemographic information, is provided in Table S2.

The characteristics of the participants—including age, gender, education level, and type of amputation—had a significant impact on the experiences of both lower-limb amputees and their family caregivers. Older amputees, particularly those who underwent transfemoral amputations, encountered greater challenges related to mobility and dependence, often exacerbated by issues such as limited walking capacity, poorly fitted prostheses, pain, and comorbidities [24]. Most caregivers were women over the age of 60, a demographic known to face heightened emotional and physical burdens in caregiving roles [25]. The lower educational attainment of both amputees and caregivers may have further restricted their ability to access and navigate available support services, contributing to a diminished quality of life for caregivers [26]. These demographic and relational factors—especially the close, often spousal, connection between dyad members—shaped caregiving dynamics, influenced coping mechanisms, and played a key role in the emergence of the study’s core themes.

From the content analysis of the interviews, four subcategories emerged: (i) difficulties faced, (ii) home discharge, (iii) the impact of amputation, and (iv) empowerment strategies. The categories and subcategories identified in the content analysis of the interviews are summarized and presented in the tables.

### 3.3. Categories and Subcategories

(i) Category I—Difficulties faced

This category highlighted the difficulties faced by both amputees and their family caregivers after amputation, revealing four key subcategories (Table 1). *Autonomy and Mobility* encompass the loss of independence and mobility, the need to adapt to a prosthesis, difficulties with balance, and the fear of falling, affecting both amputees and caregivers. *Architectural Barriers and Home Adaptation* address obstacles in the home and community, emphasizing the importance of adapting to the living environment to improve accessibility and safety. *Self-care* focuses on the difficulties amputees and caregivers face in their daily self-care tasks, including personal hygiene and household activities. *Transition and Adaptation to the New Context* highlights the lack of caregiver preparation, stressing the need for training and skill development to provide adequate support and self-care training.

The challenges faced by lower-limb amputees and their family caregivers are multifaceted, impacting various aspects of daily life and well-being. In this study, Category I explores these difficulties through four key subcategories. These subcategories reflect the key challenges of the post-amputation life, emphasizing the importance of support and adaptation to enhance the well-being of both amputees and their family caregivers. Lower-limb amputees face significant challenges with autonomy and mobility, as the loss of independence greatly limits daily activities. This subcategory includes loss of autonomy and mobility, the need for adaptation to the prosthesis, loss of postural balance, and fear of falling, which affects both amputees and caregivers. Fewer than 50% of patients retain walking ability after amputation, making it a major cause of disability [27]. Many have become dependent on assistive devices, such as crutches or wheelchairs [28]. Age and the level of amputation further affect physical balance, prosthesis satisfaction, and daily functioning, with older adults and transfemoral amputees facing the greatest challenges [29]. They also encounter physical, emotional, and practical difficulties that require support to manage mobility issues, emotions, and expectations of autonomy [7].

*Architectural Barriers and Home Adaptation* highlight significant accessibility challenges faced everyday by the amputees and often increase caregivers’ burden. Individuals with lower-limb amputations encounter various barriers to community participation and physical activity. These obstacles include navigating challenging terrains, crowded environments, and adverse weather conditions [30]. Customizing homes to address the specific needs of people with disabilities can significantly improve their autonomy and overall well-being [31]. Additionally, *Self-care* is a key subcategory; difficulty in self-care further underscores the dependency on caregivers for daily activities, intensifying emotional and physical strain. People with lower-limb amputations face various daily challenges, including mobility limitations, self-care difficulties, and social restrictions [28]. Caregivers of dependent individuals at home also encounter numerous challenges and demands, such as assisting with hygiene, feeding, mobility support, bathing, dressing, and medication management [32,33]. Many caregivers express the need for guidance on basic care, health education, and specialized training [32].

Finally, *Transition and Adaptation to the New Context* illustrates the psychological and social adjustments required to navigate a new way of living in the return home for amputees and family caregivers. Individuals with lower-limb amputations undergo a profound transition as they shift from a state of health to one of illness, confronting the loss of a limb and the resulting changes in their abilities. This adjustment involves not only physical adaptation but also the need to redefine their identity and social roles [34]. Likewise, family caregivers experience significant changes as they take on new responsibilities, necessitating the development of new skills and behaviors. This transition is often marked by emotional challenges such as grief, isolation, and anxiety, which are shaped by the availability of social support and complicated by stigma and emotional burden [35,36,37].

(ii) Category II—Home discharge

In this category, the challenges of home discharge for amputees and their family caregivers highlight the need for thorough preparation and support during this transition. The category and its, two key subcategories, as presented in Table 2 address the essential aspects of this process. Functional Training and Empowerment focus on equipping amputees with crucial skills for mobility, balance, and prosthesis adaptation, while also providing self-care training for both amputees and family caregivers, along with specialized guidance in stump care and mobilization. Family and Community Support emphasize the vital role of emotional and practical assistance from family, friends, and community organizations, while acknowledging the gaps in home support that impact both amputees and caregivers. Together, these subcategories promote the patient’s long-term autonomy, safety, and well-being.

The Home Discharge category is crucial for a patient’s recovery, involving both medical care and a holistic approach. The subcategories of *Functional Training and Empowerment* and *Family and Community Support* are key to ensuring a smooth transition. Functional training for amputees aims to restore mobility, balance, and strength, enabling them to resume daily activities. This type of training is particularly effective in improving the physical condition and functionality of lower-limb amputees. Exercise programs that combine muscular resistance and functional activities have been shown to benefit cardiorespiratory fitness, muscle strength, and overall functionality in unilateral amputees [38]. Empowering amputees and their family caregivers involve training in self-care and caregiving skills. Self-care training helps amputees perform essential tasks independently, improving their knowledge and practices [39]. Caregiver training provides family members with essential information about the condition and equips them with practical skills, such as problem-solving and strategies to manage the emotional challenges of caregiving [40].

*Family and community* support are essential sources of emotional and practical assistance. Families play a crucial role in the care of individuals with disabilities, and greater familial support for caregivers is linked to a more positive and effective perception of their caregiving role [41]. Additionally, support from associations and the broader community is vital, as it provides resources, information, and support networks for those involved. Community support has a significant positive impact on family resilience and the mental health of caregivers. Studies have identified a connection between community support and family resilience, with caregiver mental health playing a key mediating role in this relationship [42].

(iii) Category III—Impact of amputation

The impact of amputation extends beyond physical challenges, affecting both amputees and their family caregivers on the psychological, emotional, and social levels. The category and its three key subcategories, as outlined in Table 3, highlight the difficulties. *Awareness and Acceptance* involve the process of coming to terms with amputation, both for amputees and caregivers, while also recognizing the caregiver’s role and understanding the amputee’s health condition. *Psychological and emotional impact* addresses the emotional toll of amputation, including social isolation, psychological distress, and the burden placed on caregivers, thus emphasizing the need for mental health support. *Changes in Lifestyle and Routines* explores the disruptions in daily life, such as shifts in family dynamics, caregiver overload, and the emotional and social adjustments required to navigate the new reality. These subcategories illustrate the profound impact of amputation, highlighting the importance of awareness, psychological support, and adaptation strategies in enhancing the well-being of both amputees and their family caregivers.

Amputation is a significant event that impacts various aspects of an individual’s life, requiring not only physical adaptation but also a profound emotional and psychological transformation. In the context of awareness and acceptance, the amputee and their family caregiver face challenges related to social perception and understanding of their new reality, which can influence their self-esteem and social integration. This subcategory encompasses acceptance of amputation (by both amputees and caregivers), awareness of the caregiver’s role, and understanding the amputee’s health condition. According to Meleis’ Transition Theory [34], the amputee and their caregiver form a dyad experiencing a significant transition marked by adaptation to new physical, emotional, and social realities. The amputee shifts from a state of health to illness, facing the loss of a limb and changes in abilities, requiring a redefinition of identity and social roles. Simultaneously, the caregiver undergoes a situational transition, adapting to unexpected responsibilities. This dyad faces the challenge of accepting the new condition and achieving social reintegration, influenced by personal, community, and social factors. Successful transitions depend on social connections, interaction, self-confidence, and effective coping strategies. Early identification of the changes and consideration of factors that can facilitate or hinder adaptation are essential [34].

Additionally, the psychological and emotional impact is undeniable, as the loss of a body part can lead to feelings of grief, frustration, anxiety, and even depression. Amputation significantly impacts both the individuals and their caregivers, playing a crucial role in their emotional adjustment. This often leads to psychological challenges, including anxiety, depression, identity issues, low self-esteem, and social isolation. Lower-limb amputation, in particular, has profound psychological and social effects, frequently resulting in emotional distress, body image concerns, and social isolation [43,44]. Families, recognizing the difficulties faced by the amputee, often feel an increased responsibility to provide support. This shared involvement leads them to confront the emotional consequences of amputation together, experiencing a wide range of emotional reactions [45].

Along with these factors, changes in lifestyle and daily routines become inevitable, requiring adjustments in everyday activities, work, and social interactions. Lower-limb amputees face numerous challenges, often leading to social disconnection and reduced quality of life. Approximately one-third of those amputated due to vascular causes experience high levels of social isolation, negatively impacting their mental health [46]. Caregivers also face significant lifestyle adjustments, including setting aside their own routines to meet caregiving demands. They navigate conflicting emotions, use coping strategies, seek formal and informal support, and often rely on spirituality for strength [47].

(iv) Category IV—Empowerment strategies

The category *Empowerment strategies* highlights key approaches to supporting amputees and their family caregivers in areas such as psychological well-being, skill development, and social integration. This category and its six subcategories, as presented in Table 4, illustrate these strategies. *Psychological and emotional support* emphasizes the need for mental health services and peer support groups to help amputees and caregivers share experiences and cope with challenges. *Skills development and training* focuses on providing education, functional training, and self-care guidance to enhance independence and caregiving abilities. *Home visits and multidisciplinary* support highlights the importance of professional home visits and interdisciplinary care teams to ensure continuous assistance. *Technical support and accessibility* addresses the need for assistive devices, telephone support, and home adaptations to improve accessibility and safety. *Integration between hospital and community* underscores the significance of coordinated care between healthcare facilities and community services, including specialized rehabilitation centers and extended hospitalizations when necessary. *Social and economic support* acknowledges the financial and social challenges faced by amputees and caregivers and advocates for economic assistance and inclusion in community rehabilitation programs. Together, these strategies reflect the essential measures to facilitate post-amputation adaptation, fostering greater independence and resilience.

The final category that emerged from our findings was *Empowerment Strategies*, which aim to enhance the well-being and autonomy of amputees and their caregivers through comprehensive support measures. *Psychological and emotional support* play a crucial role in helping amputees and their family caregivers cope with the emotional impact of limb loss and promote overall well-being. This subcategory includes psychological and mental health support for both amputees and caregivers as well as the creation of support and peer groups for sharing experiences. The adaptation process for amputees is influenced by factors such as emotional state, social support, and the quality of the patient–caregiver relationship [48]. Additionally, psychological and mental health support for caregivers is crucial in enhancing their ability to manage the emotional and psychological challenges of daily care. Supportive interventions, such as psychoeducation, skill training, and counseling, can reduce caregiver burden while improving well-being and the overall quality of life [49].

*Skills development and training* equip them with essential competencies in daily living. Self-care training, which focuses on developing skills for self-care activities and enabling amputees to become more independent, has also proven effective in enhancing the knowledge and practices of adults with lower-limb amputations and prostheses [39]. Additionally, intervention programs can help reduce caregiver burden across various health conditions. For example, psychoeducational interventions for caregivers of hemodialysis patients have been shown to positively impact both caregiver burden and quality of life [50]. *Home visits and multidisciplinary support* provide personalized and holistic care, ensuring continuity and effective adaptation. Conducting a home visit is essential for assessing the needs of the amputee’s living environment and for providing the caregiver with appropriate resources. It helps to identify necessary home adaptations, empowers the caregiver, and ensures access to suitable assistive devices [51]. The involvement of a multidisciplinary support team ensures an integrated and holistic approach to care. According to Køberl et al. [52], implementing an integrated care model with home visits and a multidisciplinary team improves care continuity and patient safety for lower-limb amputees transitioning from hospital to home.

*Technical support and accessibility* are essential for promoting mobility and independence, particularly for older adults and people with disabilities like lower-limb amputees and helping caregivers as well. This subcategory includes the provision of assistive and alert devices, telephone support, and home adaptations to enhance accessibility. Assistive technologies such as devices and equipment that facilitate care are essential for enhancing independence and safety, particularly for older adults and people with disabilities. These technologies range from simple devices, such as portable equipment, to more complex robotic accessories that support various aspects of daily life, including mobility, education, and rehabilitation [53].

*Integrating hospital and community* care is essential to ensure a seamless transition from medical treatment to daily life. This coordination supports continuity of care during the transition from hospital to home, promoting safety and efficiency [54]. Effective communication and collaboration among healthcare professionals, amputees, and caregivers are crucial for navigating this complex transition process [54]. Social and economic support is essential for reducing financial burdens and promoting social inclusion, thereby enhancing the quality of life. For amputees, strong social support from family, friends, and community significantly improves well-being and resilience [55]. To sustain long-term care, fair economic compensation and social security benefits should be integrated into innovative and sustainable welfare policies to better support caregivers [56].

This study has some limitations that should be considered when interpreting the findings. First, it was conducted in a single vascular surgery unit in northern Portugal, which may limit the generalizability of the results to other regions or healthcare settings. Additionally, although semi-structured interviews allowed for in-depth exploration, they may have introduced interviewer bias. Another limitation is the self-selection of participants, which may have introduced bias by favoring dyads who were more willing or comfortable sharing their experiences. This could have restricted the diversity of perspectives, particularly from dyads with more conflicted or less communicative relationships. Finally, the cross-sectional design restricted the ability to establish causal relationships.

Future research should include multisite studies with longitudinal designs, focusing on the experiences of family caregivers and lower-limb amputees. Longitudinal studies would provide valuable insights into how caregiving dynamics and the experiences of both family caregivers and lower-limb amputees evolve over time, offering a deeper understanding of the long-term effects of caregiving on both parties. Expanding the participant pool to include family caregivers and lower-limb amputees from diverse socioeconomic and demographic backgrounds would strengthen the findings, ensuring that the experiences of both amputees and caregivers are fully captured, thereby enhancing the generalizability of the results.

In addition to advancing academic understanding, future studies should also explore how the findings can be effectively translated into clinical practice. This includes developing and testing strategies that promote the empowerment of both lower-limb amputees and their family caregivers, particularly through their integration into rehabilitation and follow-up care plans. Emphasis should be placed on creating family-centered empowerment programs that address the specific needs of the dyad, fostering collaboration, emotional resilience, and shared decision-making.

A holistic approach to post-amputation care is essential for addressing physical, psychological, social, and environmental challenges. Tailored functional training, emotional support, and peer groups should be provided for lower-limb amputees and their family caregivers. Effective collaboration between hospitals and community services ensures seamless care transition. Prioritizing home adaptations, assistive devices, and social and economic support can enhance accessibility, independence, and quality of life, while reducing caregiver burden and improving outcomes for amputees. These initiatives can inform policy development, guide the design of integrated care pathways, and help allocate resources more effectively to support families throughout the care journey.

## 4. Conclusions

This study highlights the complex challenges faced by amputees and their caregivers and emphasizes the need for comprehensive support that addresses the physical, emotional, and social aspects of post-amputation care.

Family-centered empowerment strategies, including caregiver education, psychological support, and enhanced accessibility, are essential for promoting resilience, autonomy, and quality of life. Effective functional training and community integration support independence and well-being.

Coordinated care between hospital and community services, along with tailored interventions, such as home adaptations and assistive devices, can significantly enhance outcomes. These findings underscore the importance of integrated hospital-to-community programs and holistic multidisciplinary strategies for a smooth transition to everyday life.

## Figures and Tables

**Table 1:** Category I—Difficulties faced.

Subcategories	Unit of Analysis
Autonomy and Mobility	“*Everything was difficult because I couldn’t do anything, I could only lie down, (…) I had difficulty walking, going to the bathroom, it was my son who carried me.*” (A17) “*Since my father was amputated, he has never done anything on his own. He can do anything, but someone has to be with him. Even if it’s just to watch.*” (FC3)
Architectural Barriers and Home Adaptation	“*I didn’t have a ramp to go to the bathroom, I had to hold onto the walker, my wife would bring a chair, and I would sit in the bathroom and then do my things. Those were the only difficulties because I didn’t have the little ramp, and the house wasn’t adapted.*” (A16) “*The bath, I had to set up the bathroom properly, so the commode and the seat chair would fit. I have all those things … I bought a shower chair. I made a ramp, which was for the wheelchair.*” (FC14)
Self-care	“*The bath was the hardest part. My daughter would bathe me in my room, just like they did in the hospital—rinsing with water, applying cream, and handing me the sponge. It’s still my daughter who helps me bathe, making sure I am clean and taken care of.*” (A20) “*My main challenge was hygiene. And then … adjusting things at home. But hygiene was the hardest part. The biggest issue was positioning—she didn’t have much strength. So, at first, I used wet wipes and would clean her in bed. Over time, I got the hang of it.*” (FC20)
Transition and adaptation to the new context	“*At the beginning, everything was difficult, bathing was hard, but I had to get used to it with the help of my family. It is very important to be taught how to take a bath.*” (A29) “*What is very important is that people should be trained and taught how to help a bedridden patient get up. I felt a lack of more precise information on how to deal with this type of patient. I was always the one who got him out of bed. My husband and I were the ones who placed him in the wheelchair, and we learned how to do it on our own.*” (FC1)

**Table 2:** Category I—Home discharge.

Subcategories	Unit of Analysis
Functional training and empowerment	“*After leaving the hospital, I went to physiotherapy, and there I was able to rebuild the muscle mass needed to use the walker again. Now, I walk with it at home from time to time.*” (A21) “*I think what’s important is the training, the hands-on practice. Watching how it’s done. It would have been helpful if someone could have come to our house to help us practice—things like bathing and mobility.*” (FC28)
Family and community support	“*When I returned home, it was my wife who helped me, and mentally, it was my son. Knowing that he accepted the situation well and was there to help made a big difference. My wife is essential in this whole process.*” (A34) *We have support from our family, and we have neighbors and friends nearby, just like now, when we need help. They are always ready to assist. We live in a more rural area, with small villages.* (FC20)

**Table 3:** Impact of amputation.

**Subcategories**	**Unit of Analysis**
Awareness and Acceptance	“*When I was told that I would have to undergo amputation, I thought to myself,* “*It has to be done, it has to be, and I have to live with it.*” *I gradually adapted and continued doing my things.*” (A25) “*I feel the obligation to take care of him, to help him, that’s what I do. Helping him also helps me a lot. But now he needs me to remove his prosthesis, to put it on, to wash it, and to help him take a bath.*” (FC29)
Psychological and Emotional impact	“*I don’t know, being stuck here feels like another illness to me. I should be out there, keeping myself distracted like I used to. But I’m here from morning to night, from here to the bed, from the bed to the chair. It makes me sad, really sad.*” (A32) “*Sometimes the caregiver needs a kind word more than the patient. Because the patient is well taken care of, but no one takes care of the caregiver. No one does anything for me. It’s not about the work; it’s about my mental state, my mind. My mind, because no one cares, because I’m the one fighting, I’m the one working, I’m the one doing everything.*” (FC18)
Changes in Lifestyle and Routines	“*Since this happened to me, I don’t go outside. It’s my daughter who forces me to go out because I just don’t feel like it.*” (A1) “*The changes… They became harder for me. I wasn’t used to being so tied down. I had my job. I had my housework and all that. But the job I had seemed to give me some relief. Now I feel more stuck at home.*” (A14)

**Table 4:** Category IV—Empowerment strategies.

Subcategories	Unit of Analysis
Psychological and emotional support	“*We should have support for these things because many people end up psychologically affected. When someone loses a leg, they’re never really the same; they’re always a bit affected mentally, and support is needed.*” (A11) “*It affects you a lot psychologically, doesn’t it? It affects the patient and even the family. That’s why having support is essential.*” (FC3)
Skills development and training	“*They should teach how to go to the bathroom and how to use the wheelchair to get to the bathroom. I needed more support, explanations on how to do things at home.*” (A16) “*There should be skills training for caregivers, like I mentioned earlier about giving a bath, but also for other things that need preparation and are necessary.*” (FC36)
Home visits and multidisplinary support	“*There needed to be a multidisciplinary team at the hospital to handle this and provide follow-up, not just in terms of direct physiatric, but also from the area we’re working in, to give some support for what could be a prosthesis.*” (A34) “*But the most important thing … for me, was having a nurse or a physiotherapist visit. That was the most important. And it would help those who care for you. But for me, in my opinion, I think it should be home care with follow-up.*” (FC34)
Technical support and accessibility	“*People, when they go home, should have the right to a wheelchair, crutches, and a prosthesis, because that matters a lot too.*” (A19) “*For example, if you don’t have an adjustable bed and lack the proper conditions, someone should provide one. If they don’t have a wheelchair and need one, someone should arrange for it.*” (FC1)
Integration between hospital and community	“*When leaving the hospital, the doctor or someone knowledgeable about the matter should call the health center to inform them that the patient is leaving and that continuity of care is needed at their home.*” (A21) “*There should be a brochure, or someone from the health center should visit the home to see how things are going, or even someone from the hospital. Someone should go to the house to check if the person has the conditions they need and then see if they really require support.*” (FC2)
Social and economic support	“*There is also the financial problem for families. I already feel that I’m spending more money now. There should be a place for people with low or weak income. Because the support from the parish council is useless.*” (A18) “*In other words, if there isn’t a social service, there should at least be some financial assistance so that people could pay someone to help care for the amputee.*” (FC15)

## Data Availability

Data from this study are available upon request from the corresponding author.

## Supplementary Materials

The following supporting information can be downloaded at: https://www.mdpi.com/article/10.3390/nursrep15050166/s1, Table S1: Characterization of amputees; Table S2: Characterization of family caregivers.

## Public Involvement Statement

No public involvement in any aspect of this research.

## Guidelines and Standards Statement

This manuscript was drafted against the consolidated criteria for reporting qualitative research (COREQ) checklist.

## Abbreviations

The following abbreviations are used in this manuscript:

ADLs	Activities of daily living
LLA	Lower-limb amputation
PAD	Peripheral arterial disease
