# Supplementary material for: Lower-Limb Amputees and Family Caregivers: Challenges, Needs, and Strategies for Empowerment—A Qualitative Study
Source: Nurs Rep. 2025 May 12;15(5):166. doi: 10.3390/nursrep15050166 (PMC12114097; doi:10.3390/nursrep15050166)
Supplement: Supplementary file 1 [file nursrep-15-00166-s001.zip › nursrep-3534709-supplementary.pdf]

**Table S1. Characterization of amputees.**

| Variables | Participants (N) | Percentage (%) |
|---|---|---|
| **Gender** | | |
| Male | 30 | 81 |
| Female | 7 | 19 |
| **Age** | | |
| 46-50 | 1 | 2,7 |
| 51-55 | 1 | 2,7 |
| 56-60 | 1 | 2,7 |
| 61-65 | 6 | 16 |
| 66-70 | 9 | 24 |
| 71-75 | 4 | 11 |
| 75-80 | 10 | 27 |
| ≥ 81 | 5 | 13,5 |
| **Education Level** | | |
| Without education | 1 | 2,7 |
| 4 years (1st stage Basic Education) | 26 | 70 |
| 6 years (2nd stage Basic Education) | 3 | 8,1 |
| 9 years (3rd stage Basic Education) | 3 | 8,1 |
| 12 years (secondary Education) | 3 | 8,1 |
| Graduate | 1 | 2,7 |
| **Employment Status at time of surgery** | | |
| Active employee | 2 | 5,4 |
| Unemployed | 1 | 2,7 |
| Retired | 20 | 54 |
| Early retired | 14 | 37,8 |
| **Amputation Level** | | |
| Transfemoral amputation | 28 | 75,7 |
| Transtibial amputation | 9 | 24 |

**Table S2. Characterization of family caregivers.**

| Variables | Participants (N) | Percentage (%) |
|---|---|---|
| **Gender** | | |
| Male | 30 | 81 |
| Female | 7 | 19 |
| **Age** | | |
| ≤ 40 | 2 | 5,4 |
| 46-50 | 3 | 8,1 |
| 51-55 | 2 | 5,4 |
| 56-60 | 4 | 11 |
| 61-65 | 2 | 5,4 |
| 66-70 | 11 | 29.7 |
| 71-75 | 5 | 13,5 |
| 75-80 | 1 | 2,7 |
| ≥ 81 | 1 | 2,7 |
| **Education Level** | | |
| Without education | | |
| 4 years (1st stage Basic Education) | 17 | 46 |
| 6 years (2nd stage Basic Education) | 4 | 11 |
| 9 years (3rd stage Basic Education) | 6 | 16 |
| 12 years (secondary Education) | 3 | 8,1 |
| Graduate | 7 | 19 |
| **Employment Status at time of surgery** | | |
| Active employee | 15 | 40,5 |
| Unemployed | 5 | 13,5 |
| Retired | 12 | 32 |
| Early retired | 5 | 13,5 |
| **Relationship with the amputee** | | |
| Spouse/partner | 24 | 24 |
| Offspring | 5 | 13,5 |
| Brother /sister | 4 | 11 |
